# Current Concepts of Cervical Spine Alignment, Sagittal Deformity, and Cervical Spine Surgery

**DOI:** 10.3390/jcm13051196

**Published:** 2024-02-20

**Authors:** Hidenori Suzuki, Masahiro Funaba, Kazuhiro Fujimoto, Yusuke Ichihara, Norihiro Nishida, Takashi Sakai

**Affiliations:** Department of Orthopedics Surgery, Graduate School of Medicine, Yamaguchi University, Yamaguchi 755-8505, Japan; mfunaba@yamaguchi-u.ac.jp (M.F.); kafuji@yamaguchi-u.ac.jp (K.F.); icchi417@yamaguchi-u.ac.jp (Y.I.); nishida3@yamaguchi-u.ac.jp (N.N.); cozy@yamaguchi-u.ac.jp (T.S.)

**Keywords:** cervical spine alignment, degenerative cervical myelopathy, cervical spondylotic myelopathy, laminoplasty, posterior fusion

## Abstract

There are not many reports on cervical spine alignment, and only a few analyze ideal surgical approaches and optimal amounts of correction needed for the various types of deformity. We comprehensively reviewed the present literature on cervical spinal deformities (with or without myelopathy) and their surgical management to provide a framework for surgical planning. A general assessment of the parameters actually in use and correlations between cervical and thoracolumbar spine alignment are provided. We also analyzed posterior, anterior, and combined cervical surgical approaches and indications for the associated techniques of laminoplasty, laminectomy and fusion, and anterior cervical discectomy and fusion. Finally, on the basis of the NDI, SF-36, VAS, and mJOA questionnaires, we fully evaluated the outcomes and measures of postoperative health-related quality of life. We found the need for additional prospective studies to further enhance our understanding of the importance of cervical alignment when assessing and treating cervical deformities with or without myelopathy. Future studies need to focus on correlations between cervical alignment parameters, disability scores, and myelopathy outcomes. Through this comprehensive literature review, we offer guidance on practical and important points of surgical technique, cervical alignment, and goals surgeons can meet to improve symptoms in all patients.

## 1. Introduction

The complex system of joints that make up the cervical spine permits the greatest range of motion of the spine and also supports the head. Many disorders and complications can occur that can lead to misalignment. Cervical spine abnormalities can cause debilitation and adverse effects that impact a patient’s overall function and health-related quality of life (HRQOL) [1]. The cervical spine is also pivotal in influencing global subjacent spinal alignment and pelvic tilt, which reflect compensatory changes needed to maintain a horizontal gaze. Multiple publications over the last 10 years have identified important radiographic parameters in the thoracolumbar spine that directly affect surgical outcomes and HRQOL, and additional studies have reported critical threshold values and global and regional parameters that aid in sagittal surgical realignment [1,2,3,4].

However, only a few publications have defined these normative values for cervical alignment, and fewer still have evaluated the influence on cervical spine surgery outcomes of segmental, regional, and global balance in a direct manner. A better understanding of cervical deformities and advancements in surgical tools and neuro-monitoring techniques have increased the number of complex cervical spine procedures being performed [3,4,5,6]. To permit a comprehensive description of the deformities, potentially achieve optimal surgical outcomes, and avoid unneeded fusions, a clear understanding of the reciprocal compensatory mechanisms occurring with such deformities is required in a clinical setting [2,3,7].

Several classification systems had reported on rigid cervical kyphosis and cervical spine deformity from the International Spine Study Group and the CSRS-Europe multi-center study project [2,8,9,10]. The classification system included a deformity descriptor and five modifiers that incorporated sagittal, regional, and global spinopelvic alignment and neurological status [8]. Koller et al. reported that the classification of cervical sagittal balance is in accord with the shape of the cervical and cervicothoracic spine, the cervical balance measured, and measures of global balance [9]. Kim et al. also revealed the classification of flat neck, focal deformity, and cervicothoracic deformity in cervical deformity [10].

However, classifications of cervical deformity are not fully established in surgical indications for degenerative cervical myelopathy (DCM), nor are the treatment options defined and clarified [2,8,9,10].

Currently, surgical indications in DCM for the correction of cervical alignment remain poorly defined, and few set standards exist regarding the amount of surgical correction needed.

This review article aims to provide a comprehensive narrative review of cervical alignment parameters and values of normal and pathological cervical alignment and to improve the understanding of the association of alignment with cervical deformity and myelopathy after cervical surgery. With this knowledge, we discuss applicable surgical strategies and the management of patients with cervical deformity, along with outcomes in light of HRQOL measures and postoperative complications. Finally, we suggest a set of pre- and postoperative guidelines on surgical management to ensure that the best results are achieved.

## 2. Degenerative Cervical Myelopathy (DCM)

**Figure 1:**
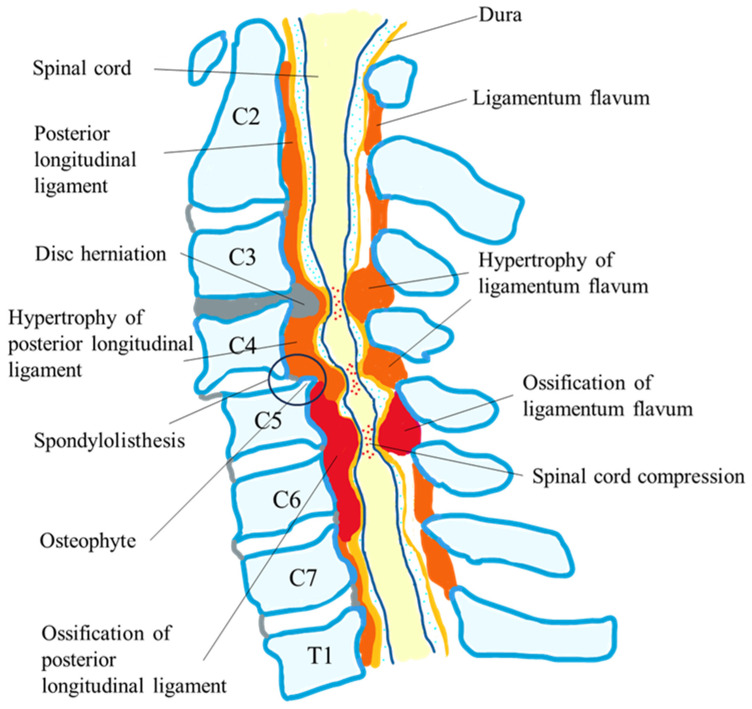
Pathology of degenerative cervical myelopathy.

DCM results when age-related osteoarthritic changes narrow the cervical spinal canal and cause chronic compression of the spinal cord and spinal nerves and subsequent neurologic disability [7,11,12,13,14,15] (Figure 1). The regional incidence for DCM was estimated to be 76 per million in North America, 26 per million in Europe, and 6 per million in Australia. In addition, the prevalence of DCM was estimated to be 1120 per one million people in Canada in 2017, with hospitalizations occurring at four per 100,000 person-years [14,15,16,17]. Patient presentation varies widely, showing symptoms from mild dysfunction, such as numbness or problems with dexterity early on, that progress to severe dysfunction, such as quadriparesis and incontinence [7,11,12,13,14,15,16,17]. Importantly, paresthesia of the extremities, which is frequently the first sign of DCM, can be easily overlooked by patients and healthcare staff because of its mildness. When a cervical disk collapses, it causes posterior bulging that narrows the spinal canal and compresses the spinal cord at that level. The resulting decrease in disk height shortens the spinal column, which ultimately causes abnormal spinal mechanics [15,16,17]. The further contribution of these altered mechanics to osteoarthritic and osteophytic changes may worsen the narrowing. Also causing compression are thickening and bending of the ligamentum flavum anteriorly toward the spinal cord. Ossification of the posterior longitudinal ligament (OPLL) also contributes to DCM by directly compressing the cord anteriorly [7,11,12,13,14,15,16,17]. Even in the presence of advanced diagnostic imaging, plain radiographs are valuable initial radiological evaluation tools of DCM. Anteroposterior and lateral radiographs show changes in the facet joints, instability, vertebral body morphology, osteophytes, abnormal ossifications, and disc space narrowing. A congenitally narrow spinal canal is also proposed as a risk factor for myelopathy in DCM (C5 canal < 13 mm). MRI is the most useful tool for the diagnosis of DCM because it allows for the assessment of vertebral bone changes, intervertebral disc degeneration, structural deformities, and the visualization of spinal cord morphology. Spinal cord signal changes in sagittal T2-weighted MR imaging are the result of pathophysiologic changes at the cellular level. Several studies have shown that the presence of spinal cord signal changes in preoperative MR imaging is associated with advanced severity of myelopathy and poor surgical outcomes [14,15,16,17].

Paresthesia in one or more extremities is the most common initial presentation in patients with DCM. They may also report decreased dexterity or “clumsiness” with buttons and zippers, changes in penmanship, changes in mobility, or frequent falls [18]. Neurological and imaging findings are important in the diagnosis of DCM.

## 3. Cervical Kyphosis and Myelopathy

Kyphotic alignment of the cervical spine may sometimes cause myelopathy to develop by forcing the spinal cord to drape against the vertebral bodies and disc-osteophyte complexes, which induces pathological changes in the anterior cord and increases longitudinal cord tension due to tethering from the dentate ligaments and cervical nerve roots [3]. Mainly, the posterior surgical approach is used in patients with DCM who suffer multi-segment spinal cord compressions, such as in those with cervical lordosis. However, it is equally applicable in some patients with neutral or cervical kyphosis. Several articles reported that after posterior laminectomy, kyphosis could be aggravated, and deterioration of neurologic symptoms may deteriorate due to scar formation surrounding the dura matter. Post-laminectomy syndromes such as segmental instability, laminar closure, and kyphosis have also been reported as risk factors [19,20].

## 4. Cervical Spine Alignment Parameters

**Figure 2:**
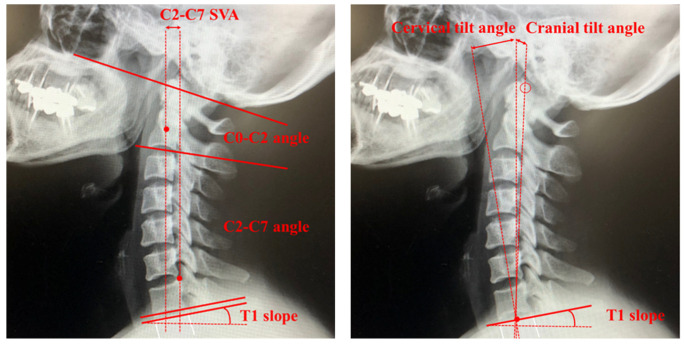
Cervical spine alignment parameters. SVA, sagittal vertical alignment.

The natural curvature of the cervical spine maintains a lordotic shape and the need to compensate for the kyphotic curvature of the thoracic spine to maintain a straight alignment of the head. The cervical alignment parameters most frequently used to assess cervical lordosis are the Cobb angles measured from C0–2 and C2–7 (Figure 2). Both C-2 sagittal vertical alignment (SVA) and C-7 SVA have been used to define sagittal alignment globally by measuring the distance between plumb lines from C-2 and C-7 (Figure 2). The slope of T1 is also a landmark of overall spinal sagittal balance and is critical in the relation between pelvic incidence and lumbar lordosis [3,21] (Figure 2). The plumb line from C-2 is especially relevant clinically because of its direct correlation with HRQOL, whereby poorer HRQOL is related to larger C-2 SVA [2,22]. The cranial tilt is defined as the angle between a line extending from the center of the T1 endplate to the tip of the dens and the plumb line (Figure 2), whereas the cervical tilt is defined as the angle between the line extending from the center of the T1 endplate to the tip of the dens and the vertical line drawn from the center of the T1 endplate (Figure 2). Chin–brow to vertical angle is an assessment of the horizontal gaze defined as the angle subtended between a line drawn from the patient’s chin to their brow and a vertical line [23]; it is especially useful when managing severe and rigid cervical kyphotic deformities because the loss of a horizontal gaze significantly affects activities of daily living and quality of life. Other parameters of cervical spine alignment parameters and definitions of their measurement are shown in Table 1.

## 5. Systematic Review of Cervical Deformity and Cervical Spine Surgery

We review the impact of sagittal malalignment in the cervical spine on surgical outcomes following DCM surgery from the articles selected in our literature search. From these same articles, we also review the various cervical spine surgical techniques that have led to better results according to the degree of cervical spine malalignment.

### 5.1. Literature Search and Inclusion Criteria

We adhered to the guidelines of PRISMA (Preferred Reporting Items for Systematic Reviews and Meta-Analyses) (https://prisma-statement.org/, accessed on 1 June 2023.) in conducting our systematic review. Using the PubMed/MEDLINE database, we initially identified relevant articles published from 1 January 2010 until 30 June 2023 that met the search terms [“Degenerative Cervical Myelopathy”, or “Cervical spondylotic myelopathy”, or “OPLL”] and [“Laminoplasty”, or “Laminectomy”, or “Spinal fusion”] and [“surgical outcome”, or “surgical result”] and [“Cervical alignment”, or “kyphosis”] and not [“Review”]. After reviewing all of the article titles, we chose titles relevant to our review. We enrolled the articles in our systematic review that included over 50 patients with cervical spine surgery. We excluded articles not written in English, and after reviewing the abstracts of obtained titles, we also excluded those articles with unrelated titles. Through the review of reference lists in the remaining articles, we identified and included additional relevant publications. Finally, we performed a full-text review of these articles and excluded those without full text.

The article selection criteria were (1) the outcome of cervical spine surgery was described, (2) the data on cervical alignment were revealed in detail, (3) the article was written in English, and (4) the number of patients in the data analysis was over 50. Our focus in this review of cervical spine alignment was on the influence of cervical malalignment on surgical outcomes.

### 5.2. Study Selection

**Figure 3:**
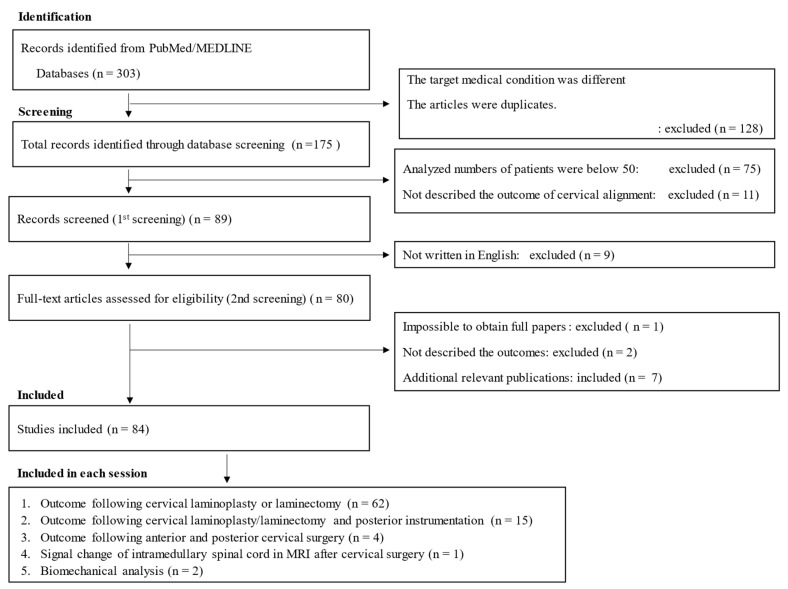
Flowchart listing the screening process for this systematic review.

Our PubMed search identified 303 potential articles. After reviewing the titles and removing duplicates, we excluded 128 articles. The abstract and full text of the remaining 175 articles were reviewed, and an additional 86 articles were excluded. These studies were excluded for the following reasons: (1) review articles, (2) only protocol papers, (3) treatment outcomes not described in detail, and (4) not suitable after discussion among the reviewers. After a further six relevant publications were added, 84 studies met the criteria for review. The search flowchart is depicted in Figure 3. Three reviewers (H.S., K.F., and M.F.) independently screened the titles and abstracts of the studies identified by the search strategy to determine their potential relevance and read the full texts of these potentially relevant studies to evaluate them for eligibility. Disagreements were resolved by consensus, and another independent reviewer (author Y.I., N.N., or T.S.) resolved any disputes if a consensus was not reached.

### 5.3. Assessment of Quality and Risk of Bias

Three review authors (H.S., K.F, and M.F.) used the Cochrane Back Review Group “risk of bias” tool to independently assess the studies for risk of bias, and an additional reviewer, author Y.I., K.F., or N.N., helped to resolve any disagreements. Critical appraisal revealed a spread in methodological quality. Common areas of bias included a lack of use of accepted diagnostic criteria for DCM/cervical spondylotic myelopathy [CSM]/OPLL malalignment and a lack of reporting of the validity and reliability of the outcome in cervical spine surgery. Selection bias was high in all studies because none reported a random selection or consecutive recruiting of participants. Eighty-four articles were selected in the present review.

## 6. Selection of Surgical Techniques for DCM and Cervical Spine Alignment [30,31,32,33,34,35,36,37,38,39,40,41,42,43,44,45,46,47,48,49,50,51,52,53,54,55,56,57,58,59,60,61,62,63,64,65,66,67,68,69,70,71,72,73,74,75,76,77,78,79,80,81,82,83,84,85,86,87,88,89,90,91,92,93,94,95,96,97,98,99,100,101,102,103,104,105,106,107,108,109,110,111,112,113,114,115]

### 6.1. Laminoplasty Following Posterior Approach

**Figure 4:**
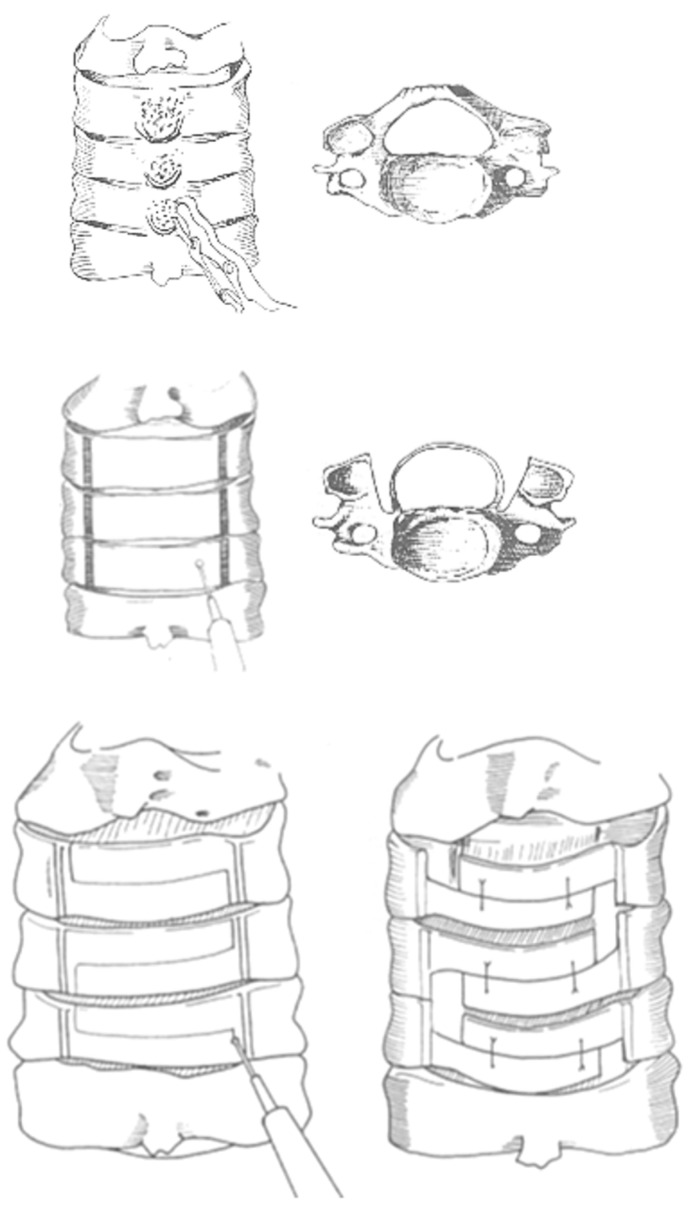
Surgical technique of Z-plasty, the origin of cervical laminoplasty. (1) Removing the spinal processes, (2) grinding and eliminating the dorsal cortical bone and cancellous bone of the laminae with an air drill, (3) incising a Z-shaped thinned laminae, leaving the ligamenta flava between the laminae intact, and (4) tying the laminae with two sutures; thus, enlargement of the spinal canal is obtained.

The 61 studies that showed a relationship between cervical alignment and the outcome following cervical laminoplasty and modified techniques are listed in Table 2. By expanding the cervical spinal canal to allow dorsal migration of the spinal cord, cervical laminoplasty can ensure indirect posterior decompression. The surgical procedures included several types of laminoplasty: z-shaped laminoplasty, open-door laminoplasty, double-door laminoplasty, muscle-sparing laminoplasty, and selective laminoplasty. In some of the laminoplasties, the surgeons used hydroxyapatite spacers and/or titanium miniplates to keep the spinous processes apart. Previous articles revealed no differences in outcomes between each surgical technique overall [34,35,36,37,38,115]. Usually, laminoplasty involves decompression of the C3–6 vertebrae either through direct decompression or by using the lordosis of the cervical spine (posterior shift of the spinal cord) as indirect decompression. Therefore, kyphotic cervical alignment causes a loss of decompression of the spinal cord and poor outcome [34,35,36,37,38,115]. Figure 4 shows the schema of the origin of laminoplasty historically performed as the Z-plasty technique reported by Hattori et al. in 1971 [34].

One of the most severe causes of DCM is OPLL, which is a hyperostotic condition that results in ectopic calcification of the posterior longitudinal ligament, as shown in Figure 1 [7]. Various papers reported that the outcome for neurological recovery after laminoplasty was poor in patients with massive OPLL or preoperative kyphotic cervical alignment compared to those with CSM due to severe anterior indentation [7,8,9,10,11,12,13,14,15].

#### 6.1.1. Cervical Spondylotic Myelopathy [38,39,40,41,42,43,44,45,46,47,48,49,50,51,52,53,54,55,56,57,58,59,60,61,62,63,64,65,66,67,68,69,70,71,72] (Table 2)

Some articles defined cervical kyphosis deformity as a C2–7 angle of ≤5° and reported that the risk factors for iatrogenic kyphosis be SVA, with a measurement of center of gravity of the head to C7 of ≥42 mm and an age of ≥ 75 years [38]. Another article revealed that postoperative cervical deformity was 13.9% at 1 year after posterior cervical laminoplasty. The risk factor for postoperative cervical deformity was decompression of C2 or C7 lamina (odds ratio [OR] = 3.1). At 1 year postoperatively, the risk factor for cervical deformity was a preoperative C2–7 SVA of ≥30 mm (OR = 19.0), and that for a C2–7 angle of ≤ −10° was a preoperative C2–7 angle of ≤2° (OR = 41.9) [38]. Abe et al. also reported that an imbalance between the T1 slope and the preoperative C2–7 angle affected the change in cervical SVA after cervical laminoplasty. If cervical SVA increases postoperatively, the O-C2 angle increases to compensate for the change and to maintain horizontal gaze [41]. Nori et al. reported that in patients with CSM, the flexion K-line may be useful as a predictor of surgical outcomes after selective laminectomy [46]. Cao et al. revealed that cephalad vertebral level undergoing laminoplasty at the C3 level and number of destroyed facet joints were also associated with kyphosis after laminoplasty in CSM patients without preoperative kyphotic alignment [56]. Focusing on other factors, Takasawa et al. revealed that among geriatric patients with CSM, preoperative malnutrition was closely associated with an increase in cervical kyphosis following laminoplasty [51].

To briefly summarize, most of the articles reported the same outcome as that below for CSM. The decreases in the angle of lordosis and ROM that occur following laminoplasty closely relate to preoperative cervical alignment. When considering the preoperative parameters for cervical alignment, the larger the patients’ capacity for extension before surgery, the smaller the amount of reduction in lordosis. In contrast, the larger the T1 slope, the greater the preoperative lordosis angle, and thus, the decrease in postoperative lordosis will also be greater in these patients.

#### 6.1.2. Ossification of the Posterior Longitudinal Ligament [73,74,75,76,77,78,79,80,81,82,83,84,85,86,87,88,89,90,91,92,93,94,95,96,97,98,99,100] (Table 2)

**Figure 5:**
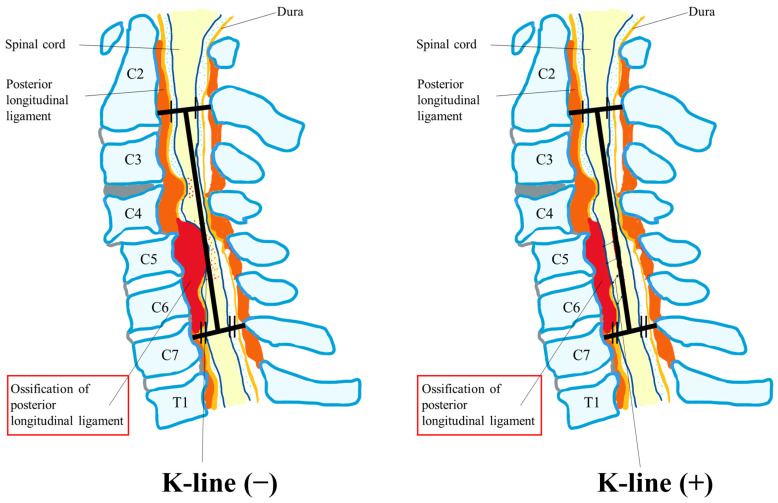
K-line.

Various papers reported poor neurological recovery after laminoplasty in patients with massive OPLL or preoperative kyphotic cervical alignment compared with those with anterior decompression with fusion (ADF) or posterior decompression with fusion (P-F) [73,74,75,76,77,78,79,80,81,82,83,84], indicating that the optimal surgical approach for cervical OPLL (C-OPLL) is still controversial. Poor surgical outcomes following laminoplasty can be influenced by several factors, and the K-line is one decision-making tool that can be used to determine appropriate surgical procedures for patients with C-OPLL. The K-line is a straight line joining the midpoints of the spinal canal at C2 and C7. Patients are K-line positive if their OPLL does not extend beyond the K-line (Figure 5). Various articles have reported a K-line and modified K-line index as a single preoperative parameter intended to account for cervical alignment and the extent of OPLL and to help predict surgical outcomes [81,83]. One article showed a K-line tilt > 20° to be a predictor of kyphotic deformity after laminoplasty in patients with K-line (+) OPLL, and laminoplasty is not suitable for these patients [81].

We reported that older age, longer disease duration, postoperative deterioration of cervical kyphosis, and a C2–7 angle during neck flexion negatively affected outcomes of HRQOL measures [99]. Kinematic computed tomography myelography and examinations of multimodal spinal cord evoked potentials revealed that the patients with the most severe spinal cord compression during neck flexion were associated with smaller cervical lordosis during neck extension and increased anterior spondylolisthesis during flexion. Patients with this type of compression were characterized neurologically by severe lower limb dysfunction [100]. We also revealed signal intensity changes on magnetic resonance imaging (MRI) that were caused by severe and poorly compensated spinal cord compression during neck flexion. A snake eye appearance may be a useful sign of inferior postoperative recovery of upper limb function, whereas multilevel high signal intensity may indicate inferior postoperative recovery of lower limb function [12].

We summarize the rate of occurrence of postoperative cervical deformity and preoperative risk factors for each parameter and other etiologies in C-OPLL in Table 2. In addition, we summarize in Table 2 the relationship between postoperative cervical malalignment and HRQOL outcome measures based on the Neck Disability Index (NDI), Visual Analogue Scale (VAS), Short Form 36 Health Survey (SF-36), and Modified Japanese Orthopaedic Association (mJOA) questionnaires.

**Table 2:** Laminoplasty for CSM/OPLL and cervical spine alignment.

1st Author	Year	Patient’s Number (CSM/OPLL)	Definition of Cervical Deformity	Type of Laminoplasty (LAMP)	Risk Factors of Postoperative Malalignment	Prediction Factors of Postoperative Malalignment before Surgery	Occurrence Rate of Postoperative Cervical Deformity	Risk Factors of Poor HRQOL Outcome
Sakai K [37]	2016	CSM: 174	-	Double-door LAMP	-	C2–7 angle ≤5° CGH-C7 SVA ≥ 42 mm Age ≥ 75 years	5.2%	Cervical JOA score was in the kyphotic deformity (+) group were inferior.
Oe S [38]	2023	CSM and OPLL: 193	SVA ≥ 40 mm C2–7 angle ≤ −10°	Laminectomy Double/Open-door LAMP	Decompression of the C2 or C7 lamina.	C2–7 SVA ≥ 30 mm C2–7 angle ≤ 2°	13.9%	No difference in post-cervical deformity
Kim BJ [39]	2021	CSM: 26, OPLL: 27	-	Open/French-door LAMP (Opening lamina was fixed with a titanium miniplate)	-	Preoperative lower extension capacity The greater T1 slope	-	-
Abe T [40]	2022	CSM: 110	-	Double-door LAMP (Hydroxyapatite spinous process pacers)	Increase in O–C2 angle	Imbalance between T1 slope and preoperative C2–7 angle	-	-
Ninomiya K [41]	2022	CSM: 732	Anterior cervical spondylolisthesis (Slippage of ≥2 mm) C2–7 angle ≤ −5°	Double/Open-door Selective LAMP	Anterior cervical spondylolisthesis with kyphosis	Anterior cervical spondylolisthesis with kyphosis	2.3%	Preoperative cervical kyphosis
Zhang H [42]	2015	CSM: 198	-	Open-door LAMP	No risk of different angles in lamina open-door	--	-	No difference in different angles in lamina open-door
Nurboja B [43]	2012	CSM: 268	-	Laminectomy Four or more cervical levels LAMP	-			
Guo Q [44]	2022	CSM: 92	-	Muscle preserved vs. conventional open-door LAMP	No difference	No difference	-	No difference
Lee DG [45]	2013	CSM and OPLL: 64	-	Open-door vs. French-door LAMP	No difference	-	-	No difference
Nori S [46]	2020	CSM: 159	-	Muscle-preserving selective laminectomy	Flexion K-line (−)	Flexion K-line (−)	-	Flexion K-line (−) group
Jain A [47]	2017	CSM: 68	-	Conventional laminectomy	-	No difference in pre-cervical lordosis		No difference in post-cervical lordosis
Liu C [48]	2023	CSM: 79	C2–7 angle ≤ −10°	Open-door LAMP (mini titanium plate system)	Low ext. ROM	Low ext. ROM and high flex ROM	-	Low ext. ROM
Fujishiro T [49]	2021	CSM: 111	-	Double-door LAMP	Gap ROM exceeding 30°	Preoperative gap ROM exceeding 30°	-	-
Lin BJ [50]	2015	CSM: 26	-	Open-door LAMP	C2–7 angle ≤ 10°	C2–7 angle ≤ 10°	-	Morphology was that of a neutral or posterior convexity-type spinal cord accompanied by kyphotic deformity
Chen HY [51]	2020	CSM: 85	Kyphosis: C2–7 angle < 0°	Spinous process splitting LAMP	-	C2–7 SVA ≥ 30 mm T1 slope ≤ 2° T1 slope minus C2–7 angle ≥ 20°	-	C2–7 SVA < 2.89 cm T1 slope minus C2–7 angle ≥ 20°
Takasawa E [52]	2023	CSM: 90 (age ≥ 65)	-	Open-door LAMP	-	Malnutrition status (Geriatric nutritional risk index < 98)	13.3%	Malnutrition status
Wang Z [53]	2021	CSM: 72	T1 Spino-cranial angle > 20°: cervical imbalance	LAMP	-	T1 Spino-cranial angle > 20°	-	T1 spino-cranial angle > 20°
Ikeda T [54]	2023	CSM/OPLL /CDH: 70	K-line (+)/(−)	Miyazaki and Kirita’s method (*n* = 66) Kurokawa’s method (*n* = 4)	K-line (−)	K-line (−)	-	K-line (−) of osseous type
Nori S [55]	2022	CSM: 81	Neck-flexed position K-line (+)/(−)	C4–C6 LAMP with C3 and C7 partial laminectomy	Neck-flexed position K-line (−)	Neck-flexed position K-line (−)	-	Neck-flexed position K-line (−)
Cao J [56]	2017	CSM: 194 (without preoperative kyphotic alignment)	-	Open-door LAMP (mini titanium plate system)	Cephalad vertebral level undergoing laminoplasty at the C3 level Facet joint destroyed	The greater T1 slope The greater C2–7 SVA	10.8%	Postoperative kyphotic deformity Cervical laminoplasty starting at the C3 level
Kurihara K [57]	2022	CSM: 178	Spondylolisthesis (slippage > 2 mm)	Selective LAMP	No risk	No risk	16.3%	No risk
Laiginhas AR [58]	2015	CSM: 57	Kyphosis development (Ishihara index: 19.3) Subclinical cervical instability.	LAMP	-	-	8.8%	-
Minamide A [59]	2017	CSM: 78	-	Microendoscopic laminotomy vs. conventional LAMP	Conventional LAMP	-	-	-
Taniyama T [60]	2014	CSM: 61	Nonlordotic: straight, sigmoid, reversed sigmoid and kyphosis type	Miyazaki and Kirita’s method LAMP	-	Minimal interval between the K-line and most anterior feature of the spinal canal	-	Minimal interval between the K-line and most anterior feature of the spinal canal
Ninomiya K [61]	2021	CSM: 379	Kyphosis: preoperative C2–C7 angle < 0°	Selective LAMP	-	Smaller C7 slope Larger C2–C7 SVA	-	-
Chen G [62]	2020	CSM: 369	C2–7 angle ≤ −5°	Open-door LAMP (mini titanium plate system)	-	Longer C2–7 SVA Larger C7 slope Smaller C2–7 angle Larger fROM More cranial operation level	7.8%	-
Machino M [63]	2020	CSM: 1025	Kyphosis: preoperative C2–C7 angle < 0°	Modified double-door LAMP	-	C2–7 angle ≤ 7°	14.7% (Preoperative C2–7 angle ≤ 7°)	-
Yang Y [64]	2023	CSM: 266	-	Open-door LAMP	-	C2–7 angle ≤ 7° C2–7 extension angle ≤ 18°	9.77%	No difference in alignment
Zhang JT [65]	2017	CSM: 41 (C2–7 angle > 5°)	-	Open-door LAMP (plate system)		Higher T1 slope Greater C2–7 SVA Starting laminoplasty at C4 level	-	-
Shimizu T [66]	2022	CSM: 120	Residual anterior spinal cord compression	Double-door LAMP	-	C3–4 level CSM: C3 partial decompression, modified K-line and segmental lordotic angle C4–7 level CSM: Segmental lordotic angle	33.3%	Residual anterior spinal cord compression
Obo T [67]	2022	CSM: 138	Anterolisthesis ≥2 mm Retrolisthesis ≥ 2 mm Translational instability ≥ 3 mm	LAMP	Greater flex ROM Smaller extension ROM	Greater flex ROM Smaller extension ROM	Anterolisthesis 8.7% Retrolisthesis 23.9% Translational instability 11.6%	-
Chang H [68]	2017	CSM: 67	-	Selective laminectomy vs. C3-6 LAMP	No difference	-	-	No difference
Sivaraman A [69]	2010	CSM: 50	-	Skip Laminectomy vs. LAMP	-	-		Skip laminectomy: better outcome
Nagoshi N [70]	2021	CSM: 80	Kyphosis: Preoperative C2–C7 angle < 0°	Expansive unilateral open-door LAMP	Smaller C7 slopes	Preoperative kyphosis	-	Preoperative kyphosis: No risk
Rao H [71]	2019	CSM: 85	T1 slope minus C2–C7 lordosis < 20°	Open-door LAMP	-	T1 slope minus C2–C7 lordosis < 20°	-	-
Machino M [72]	2018	CSM: 1025 (<65/65–74/≥75 years)	-	Modified double-door LAMP	-	Elderly patients	-	Elderly patients
Yeh KT [73]	2016	DCM: 65	-	Modified expansive open-door LAMP	-	none	-	-
Kanbara S [74]	2018	OPLL: 100	K-line (+)/(−)	Double-door LAMP	Postoperative flexion value > 0 (Flexion ROM minus extension ROM)	K-line (−)	-	K-line (−)
Kawaguchi Y [75]	2019	OPLL: 153	-	en bloc cervical LAMP	-	-	-	Kyphotic cervical alignment High-intensity lesions in the spinal cord on T2-MRI
Chen Z [76]	2016	OPLL: 819	-	Anterior corpectomy and fusion with LAMP vs. LAMP	-	-	-	Occupying ratio ≥ 60% Kyphotic alignment High cord signal on T2WI
Kim B [77]	2016	OPLL: 64	Loss of cervical lordosis	LAMP	-	Higher T1 slope Lower T1 slope minus C2–C7 angle	-	-
Liu X [78]	2019	OPLL: 132 (OPLL locating at the C2 segment)	-	C2–C7 vs. C3–7 LAMP	C2–C7 LAMP: no risk for malalignment	-	-	No difference
Nakashima H [79]	2023	OPLL: 165	Postoperative loss of cervical lordosis > 10° or >20°	LAMP	-	Preoperative small extension ROM (cutoff value: 7.4°) Large occupation ratio of OPLL (cutoff value of 39.9%)	19.4%	Postoperative loss of cervical lordosis > 20°
Li C [80]	2022	OPLL: 81	K-line (+)/(−)	Single-door LAMP	-	K-line (−) and lordosis < 7°	K-line (−): 30.77%	K-line (−)
Sakai K [81]	2023	OPLL: 62	Kyphosis: preoperative C2–C7 angle < 0°	Double-door LAMP	-	K-line tilts > 20°	6.5–9.7%	Kyphotic deformity
Yoo S [82]	2017	OPLL: 73	-	Laminectomy vs. LAMP	Laminectomy: no risk for malalignment in continuous-type OPLL	-	7.9–8.6%	Laminectomy: no risk for malalignment in continuous-type OPLL
Saito J [83]	2020	OPLL: 72	-	LAMP for K-line (+) OPLL	-	Preoperative segmental ROM at the peak of OPLL Large OPLL occupation ratio	-	Preoperative segmental ROM at the peak of OPLL
Xu C [84]	2020	OPLL: 181	-	LAMP	-	Large CGH (center of gravity of the head) minus C7 SVA Large T1 slope	-	Postoperative lordosis loss
Kim SW [85]	2020	DCM: 83	Kyphosis: Straight, sigmoid, and kyphosis groups (Toyama classification)	Double-door LAMP (Hydroxyapatite spinous process pacers)	Non-reducible ROM	Preoperative kyphosis	-	--
Shimizu K [86]	2021	DCM: 113	-	Open- or double-door LAMP (Semispinalis cervicis muscles: preservation (vs. detachment)	Detaching the semispinalis cervicis muscle	-	-	-
Chen H [87]	2015	DCM: 57	-	Open-door LAMP (Mini-plate fixation vs. Suture suspension fixation)	Open-door LAMP: Suture suspension fixation	-	-	Open-door LAMP: Suture suspension fixation
Sakamoto R [88]	2022	DCM: 100	-	Double-door LAMP	-	T1 slope minus cervical lordosis > 20°	-	T1 slope minus cervical lordosis > 20°
Qian S [89]	2018	DCM: 137	-	Open-door LAMP	Kyphosis alignment with CSM	Kyphosis alignment with CSM Preoperative ROM restriction	-	Kyphosis
Fujiwara H [90]	2018	DCM: 57	-	Open-door LAMP	-	-	-	No correlation with radiologic parameters
Kato S [91]	2021	DCM: 109	-	LAMP	Large local kyphosis angles	Small C2–7 Large local kyphosis angles	-	Large local kyphosis angles
Kim SW [92]	2013	CSM: 28 OPLL: 30	-	Double-door LAMP (Preoperative C2–C7 angle < 10° vs. ≥10°)	No risk	No risk	-	No difference
Sakaura H [93]	2011	DCM: 53	Kyphosis and lordosis were defined as C2–7 angle < 10° and ≥10°	Open-door LAMP (minimum 5-year follow-up)	No risk	No risk	-	No risk
Ebata S [94]	2015	DCM: 66	-	Double-door LAMP (Short length of rest with a cervical orthosis: 8, 4, or 2 weeks)	No risk	No risk	-	No difference
Matsuoka Y [95]	2018	DCM: 84	C2–7 SVA ≥ 80 mm C2–7 angle < 0°	LAMP	-	Small SVA with lumbar hyper-lordosis (Patients having no preoperative cervical and global spinal sagittal imbalance)	25.6%	-
Wang Z [96]	2021	DCM: 68	-	LAMP (with a plate fixation System)	-	Too high or low preoperative spino cranial angle	-	-
Kire N [97]	2019	DCM: 110	-	LAMP (Patients with C2–C7 > 10°, lordotic)	No risk	No risk	-	No risk
Kong Q [98]	2011	DCM: 76	-	LAMP (Patients with a straight or lordotic cervical spine)	-	Little space available at cephalad levels is key factor in predicting cord shift distance in laminoplasty	-	Whether the anterior indirect decompression was adequate or not.

CSM, cervical spondylotic myelopathy; OPLL, ossification of the posterior longitudinal ligament; LAMP, laminoplasty; HRQOL, health-related quality of life; SVA, sagittal vertical alignment; DCM, degenerative cervical myelopathy; ROM, range of motion.

### 6.2. Laminectomy/Laminoplasty and Posterior Fusion Following the Posterior Approach

Fifteen studies that showed the relationship between cervical alignment and outcome following posterior fusion are listed in Table 3 [101,102,103,104,105,106,107,108,109,110,111,112,113,114,116]. We summarize the pre- and postoperative risk factors for each parameter and other etiologies in the cervical spine in Table 3. We also summarize the relationship between postoperative cervical malalignment and HRQOL outcome measures based on the NDI, VAS, SF-36, and mJOA questionnaires in Table 3.

#### 6.2.1. Laminectomy and Posterior Fusion for CSM [101,102,103,104,105,106,107] (Table 3)

Several articles investigated the surgical efficacy of ADF vs. laminoplasty only vs. P-F as the treatment for CSM and further analyzed the changes in cervical spinal alignment parameters and HRQOL outcome. Satisfactory neurological improvement was obtained with all of the surgical types. However, ADF was more suitable than P-F in restricting postoperative kyphotic change and an increase in SVA, even if ADF was not suitable for more than three levels of spinal cord compression [101]. Another article revealed that the most important cervical sagittal parameters correlating with clinical outcomes were C7 slope, occipito-C2 angle, external auditory meatus tilt, and SVA and that ADF more efficiently restored cervical sagittal alignment [102]. Pain outcomes following cervical sagittal alignment were similar between the patients with laminoplasty and those with laminectomy with fusion (L-F). However, among laminoplasty patients, and especially those with lordosis greater than 20°, greater cervical lordosis was associated with better pain outcomes [104]. A different paper reported that L-F resulted in a better clinical outcome than laminoplasty alone for patients with CSM having local kyphosis exceeding 5° [105].

Surgery for CSM in the patients without kyphosis showed no significant differences between the three surgical approaches overall. However, for patients with severe local kyphosis, ADF was the better surgery for the maintenance of cervical alignment.

#### 6.2.2. Laminectomy and Posterior Fusion for OPLL and DCM [108,109,110,111,112,113,114,116] (Table 3)

Eight articles reported the surgical efficacy of ADF and/or P-F in the treatment of CSM, along with the changes in cervical spinal alignment parameters. One article concluded that laminoplasty is not useful for K-line (–) cervical OPLL but that ADF was one of the suitable surgical treatments for K-line (–) OPLL [108]. Another article showed that cervical laminoplasty and P-F provided almost comparable improvements in function and QOL two years after surgery, although the frequency of perioperative complications was greater in the patients who received P-F [109]. Lee et al. mentioned that OPLL might worsen more frequently after laminoplasty and that P-F and laminoplasty were preferable techniques for C-OPLL, with the former better for patients with high SVA distances at the baseline measurement [110]. Liu et al. recommended laminoplasty for patients with OPLL and straight cervical lordosis when taking into consideration comparable neurological recovery, less axial pain, and better improvement in neck function compared with those following P-F [111]. Moon et al. concluded that direct spinal cord decompression with ADF provides better long-term stable neurologic outcomes than laminoplasty [112]. Among the patients with DCM, overweight patients (body mass index [BMI] > 25 kg/m^2^) who underwent P-F had a greater increase in SVA than normal-weight patients, while rates of reoperation were similar. In addition, preoperative cervical lordosis increased with increasing BMI [113]. One paper revealed the levels of P-F reflecting postsurgical malalignment and the rate of revision. They concluded that multilevel posterior cervical fusions should be extended to T1 because stopping a long construct at C7 increases the rate of revision [114]. Kato et al. reported a significant association between cervical deformity and both preoperative disease severity and postoperative outcomes; however, they showed no effects of corrections of the deformities [116].

**Table 3:** Laminectomy and posterior fusion for CSM/OPLL and cervical spine alignment.

1st Author	Year	Patient’s Number (CSM/OPLL)	Definition of Cervical Deformity	Surgical Techniques (vs. Other Surgical Approach)	Risk Factors of Postoperative Malalignment	Prediction Factors of Postoperative Malalignment before Surgery	Risk Factors of Poor HRQOL Outcome
Du W [101]	2022	CSM: 117	C2–7 angle ≤ 0°	Laminectomy with posterior screw fixation (L-F) (vs. Anterior decompression with fusion, ADF)	L-F	-	No difference between ADF and L-F
Li XY [102]	2023	CSM: 167	Kyphosis: C2–7 angle < 0°	L-F (vs. ADF and LAMP)	C2–7 angle < 10°	-	-
Liu H [103]	2021	CSM: 97	-	L-F (vs. ADF)	L-F	-	Malalignment of C7 slope, occipito-C2 angle, external auditory meatus tilt, and cervical sagittal vertical axis.
Lau D [104]	2017	CSM: 145	-	L-F (vs. LAMP)	No difference between L-F and LAMP	-	L-F > LAMP
Miyamoto H [105]	2012	CSM: 60 (Local kyphosis ≥ 5°)	Local kyphosis ≥ 5°	L-F (vs. LAMP)	LAMP	-	LAMP > L-F
Ashana AO [106]	2021	CSM: 66	Kyphosis: C2–7 angle < 0°	L-F (vs. LAMP)	LAMP	--	L-F = LAMP
Lee JJ [107]	2022	CSM: 67	-	L-F (posterior cervical fusion at C5/6 with those at C7/T1.)	End of posterior cervical fusion at C5/6	-	End of posterior cervical fusion at C5/6
Koda M [108]	2016	OPLL: 48	K-line (+)/(−)	L-F (vs. LAMP)	LAMP for K-line (−)	K-line (−)	LAMP > L-F
Nakashima H [109]	2022	OPLL: 189	-	L-F (vs. LAMP)	No difference	No difference	L-F = LAMP
Lee CH [110]	2016	OPLL: 57	-	L-F (vs. LAMP)	LAMP	-	LAMP (SVA ≥ 40 mm)
Liu X [111]	2017	OPLL: 67	-	L-F (vs. LAMP)	LAMP	-	L-F > LAMP
Moon BJ [112]	2018	OPLL: 352	-	L-F (vs. ADF)	-	-	L-P > ADF High grade of MR signal intensity
Perez EA [113]	2022	DCM: 198	-	L-F	BMI > 25 with CSM undergoing P-F (greater increase in SVA)	BMI > 25 with CSM undergoing P-F (greater increase in SVA)	-
Schroeder GD [114]	2016	DCM: 219	-	L-F Multilevel posterior cervical fusions ending at C7 vs. T1 vs. T2-T4	No difference	-	Construct terminated at C7
Kato S [116]	2018	DCM: 178	C2–7 angle < −10° (kyphosis) and/or SVA > 40 mm.	L-F (vs. ADF, combine)	L-F Older age Preoperative deformity	Preoperative deformity	Post/Preoperative deformity

CSM, cervical spondylotic myelopathy; OPLL, ossification of the posterior longitudinal ligament; HRQOL, health-related quality of life; L-F, laminectomy with fusion; ADF, anterior decompression with fusion; LAMP, laminoplasty; DCM, degenerative cervical myelopathy; BMI, body mass index; SVA, sagittal vertical alignment.

### 6.3. Anterior Fusion and Posterior Decompression for CSM/OPLL *[117,118,119,120,121]*

The four studies that showed a relationship between cervical alignment and outcome following cervical laminoplasty are listed in Table 4. In patients with CSM, both procedures (ADF with laminoplasty vs. laminoplasty only) resulted in significant neurological improvements and reduction in pain. Similar results of these procedures for decompression and neurological improvement were also shown. However, surgical costs, surgical time, blood loss, and the rate of complications with the posterior approach were superior to those of the single-stage combined approach [117]. ADF combined with laminoplasty yielded an excellent outcome for CSM patients with concomitant short-segment kyphosis, instability, or major anterior pathology [118]. Sun et al. revealed that in the treatment of severe cervical OPLL, ADF achieved better recovery for spinal cord expansion, spinal cord alignment, and Cobb angle, along with better postoperative JOA scores and fewer complications compared with posterior laminectomy [119]. For patients with long-segment massive OPLL, Li et al. suggested laminectomy at the compression level followed by ADF depending on the severity and range of compression or corpectomy of not more than three vertebral levels on the same day or within 2 weeks. For K-line (–) patients with massive OPLL involving the C2, they suggest posterior laminectomy above and below the levels affected by OPLL, along with simultaneous posterior fixation [120].

### 6.4. Others

Shin et al. reported the postoperative predictors of neurologic outcome for C-OPLL that included all of the surgical approaches. They concluded that factors associated with less improvement in myelopathy in patients with worse baseline function were those that more influenced the surgical outcome rather than cervical malalignment. i.e., older age, male sex, intramedullary high signal intensity, and posterior decompression [121]. Another article mentioned the predictors of neurologic outcomes in MRI studies [122]. Yagi et al. revealed that patients with changes in intramedullary signal intensity on MR images experienced significantly worse long-term clinical outcomes. The reported risk factors were cervical spine instability and severe compression of the ventral spine. Patients with postoperative expansion of the area of high signal intensity as a risk factor also had significantly worse long-term clinical outcomes [122]. Several other articles reported that biomechanical studies revealed the relation between cervical alignment and changes in the spinal cord due to stress [123,124].

## 7. Discussion

The ideal result of cervical laminoplasty after surgery is to recover from a neurological deficit through nerve decompression brought about by effective posterior migration of the spinal cord. Previous studies showed the important role that cervical sagittal alignment plays in the clinical outcomes of laminoplasty and the posterior approach in cervical surgery [34,35,36,115]. Severe kyphotic curvature of the cervical spine is not recommended as a good indication for laminoplasty only because laminoplasty may not create adequate posterior migration of the spinal cord in cervical OPLL. Although long-term results of conventional C3–7 laminoplasty have shown significant incidences of postoperative kyphotic deformity and loss of ROM, in fact, the number of patients reported following cervical surgery is small [1,2,3,4,5,125]. In vitro cadaveric study of cervical spinal cord intramedullary pressure (IMP), it was also reported that IMP increased significantly as the cervical kyphotic deformity exceeded +21° [126]. However, high-level objective evidence as to which of these surgical approaches is superior in terms of patient outcomes and complication profiles is still lacking in the literature. The goal of surgery is to decompress the spine and spinal cord, maintain the alignment, and improve or preserve neurological function. Treatment decision-making with an anterior versus a posterior approach for multilevel DCM has been controversial and is still debated in each patient [127,128].

We showed a brief summary of the systematic review of prediction factors of postoperative malalignment before surgery, the risk factors of postoperative malalignment, and the surgical outcomes in each surgical approach. In patients with CSM/OPLL/DCM, the risk factors were preoperative kyphosis (C2–7 angle ≤ 0–10°), SVA ≥ 30–42 mm, greater T1 slope, spondylolisthesis, K-line (−), flex K-line (−), greater flex ROM, gap ROM exceeding 30°, age over 75 and large OPLL occupation ratio, etc. Surgery for CSM in the patients without kyphosis showed no significant differences between ADF, laminoplasty only, and P-F overall. However, for patients with severe local kyphosis, ADF was the better surgery for the maintenance of the cervical alignment. Poor surgical outcomes following laminoplasty can be influenced by several factors, and the K-line is one decision-making tool that can be used to determine appropriate surgical procedures for patients with C-OPLL. The optimal surgical approach for C-OPLL is still controversial. However, ADF and/or P-F are some of the suitable surgical treatments for K-line (–) OPLL with local kyphosis, large OPLL, and/or cervical kyphosis. 

The CSRS-Europe classification of sagittal cervical balance classified the alignment according to the shape of the cervical and cervicothoracic spine, the cervical balance measured (C2–C7 SVA), and measures of global balance (C7–S1 SVA) [9]. They corrected the spinal alignment by osteotomy and spinal fusion according to the classification. They reported that patients with complications had larger preoperative regional kyphosis angle (RKA) and postoperative increase in distal junctional kyphosis angle. In addition, patients with revision surgery had a larger RKA-change and postoperative translation. A total of 21% of patients had a postop segmental motor deficit, and the risk was elevated in the osteoporosis group [9]. In our systematic review, most articles focused on the advantages of preservation of cervical alignment and the outcome of operating and/or correcting alignment by ADF and/or P-F. However, this article suggested that the correction and osteotomy surgery for DCM patients is a high-risk operation and is careful with several severe complications [9]. This is important information for spine surgeons when deciding the surgical approach for DCM patients. 

Although cervical spine deformity is complex and significantly affects patient quality of life, on the basis of clinical and radiographic parameters determined by a literature review and a modified Delphi approach with an expert panel, Ames et al. created the classification system of cervical spine deformity [8]. The classification system included a deformity descriptor and five modifiers that incorporated sagittal, regional, and global spinopelvic alignment and neurological status. The descriptors included ‘C’, ‘CT’, and ‘T’ for primary cervical kyphotic deformities with an apex in the cervical spine, cervicothoracic junction, or thoracic spine, respectively; ‘S’ for primary coronal deformity with a coronal Cobb angle ≥ 15°; and `CVJ’ for primary craniovertebral junction deformity. The modifiers included C2–7 SVA, horizontal gaze (chin–brow to vertical angle), T1 slope minus C2–7 lordosis, myelopathy (Modified Japanese Orthopaedic Association scale score), and the Scoliosis Research Society (SRS)-Schwab classification for thoracolumbar deformity [8]. The recent classification system may be a novel system; however, it requires (1) full-length standing postero-anterior and lateral spine radiographs that include the cervical spine and femoral heads; (2) standing postero-anterior and lateral cervical spine radiographs; (3) a completed and scored mJOA questionnaire; and (4) a clinical photograph or radiograph that includes the skull for measurement of the chin–brow to vertical angle [8]. The requirement of this scoring system is complex, and a full-length standing spine radiograph can be taken only in a few institutions in clinical. The current literature review in this study revealed that this scoring system for DCM surgery still has difficulties being widely used as a popular classification system in clinical settings. 

Another simple classification was published by Kim et al. [10]. They classified the three types of cervical deformity: flat neck, focal deformity, and cervicothoracic deformity. The development of the classification system may guide the surgical treatment for deformity and the choice of fusion level [10].

We need to expand our education on the effects of various clinical and radiographic factors in cervical spinal surgery by using new types of classifications such as these.

Numerous factors, such as patient age, number of involved levels, prior surgical approaches, location of compressive lesions, and cervical alignment and flexibility, affect the surgical approach to addressing DCM, and this is the same for alignment parameters. Standing balance is achieved by neuro-musculoskeletal components of the entire body working together and is sustained by a “chain” comprised of key components: the feet, pelvis, and cranium [129]. From this point of view, going forward, we need to have a three-dimensional (3D) understanding of the alignment and balance of the spine and whole body, and not just cervical alignment. Hasegawa et al. used EOS™ slot-scanning 3D X-ray imaging to evaluate standing sagittal alignment of the whole axial skeleton with reference to the gravity line in humans and reported that cervical lordosis, pelvic tilt and incidence, hip extension, knee flexion, and ankle dorsiflexion were all related significantly with age. They also provided evidence that compensation in whole-body alignment, as indicated by several sagittal parameters, correlates with HRQOL scores [130,131,132]. We think that all of these parameters are linked in mutual relationships to cervical spine alignment and sagittal deformity following cervical spine surgery. We revealed the theme of this article to be the certain conclusion that changes in alignment following cervical spine surgery with each of the surgical techniques correlate with HRQOL scores. However, we may need to analyze whole-body alignment, including the lower limbs, to reach a definitive conclusion regarding cervical spine alignment, sagittal deformity, and cervical spine surgery.

Physiotherapy has also been shown to be effective in maintaining cervical lordosis [133]. Traditional spinal manipulative therapy has largely proven unsuccessful in increasing cervical lordosis. The long-term maintenance of symptomatic relief in patient groups receiving the spinal traction method, cervical extension traction (CET), as a part of the rehabilitation resulted from achieving increased cervical lordosis. This is substantiated by the fact that six trials featured the CET as the only difference between the treatment and comparison group treatment arms [133]. The combination of the surgery and the rehabilitation for DCM patients with cervical kyphosis has the potential to influence better outcomes. 

## 8. Conclusions and Outlook

Cervical sagittal alignment plays an important role in the clinical outcomes of laminoplasty and the posterior approach in cervical surgery. We comprehensively reviewed the current literature on cervical spinal deformities (with or without myelopathy) and their surgical management to create a framework on which surgical planning can be based. A general assessment was made of the parameters, and correlations between the alignment of the cervical and thoracolumbar spine were presented. We also analyzed the approaches (posterior, anterior, or combined) and techniques (laminoplasty, laminectomy, anterior cervical discectomy, and fusion) of cervical surgery and their indications. Finally, a complete evaluation of outcomes and postoperative HRQOL measures based on relevant questionnaires was addressed in this article. Most patients with DCM experienced postoperative neurologic improvement, but compared with patients with preoperative kyphotic alignment, those with preoperative lordotic alignment showed greater improvement. A correct surgical approach may also be guided by the K-line or effective cervical lordosis. Cervical alignment correction from K-line (−) to K-line (+) by posterior fixation and/or anterior fusion may lead to a more effective outcome in the surgery for C-OPLL. However, we need future research to reveal the hypothesis. Additional research incorporating prospective randomized trials should be performed to further elucidate other factors to help guide the proper management of patients with DCM and cervical sagittal malalignment.

## Figures and Tables

**Table 1:** Cervical spine alignment parameters and measurement description.

Parameter	Measurement Description	Reference
Neck tilt	The angle formed by a line drawn from the upper end of the sternum and a line connecting the center of the T1 upper endplate (UEP)	[21]
Thoracic inlet angle	The angle formed by a line from the center of the T1UEP vertical to the T1UEP and a line connecting the center of the T1UEP and the upper end of the sternum.	[24]
Cranial incidence	The angle between the center of the line perpendicular to the McGregor line and the line that joins the middle of the McGregor line to the sella turcica	[25]
Cranial slope	The angle between the horizontal line and the McGregor line	[21]
Cranial tilt	The angle between the vertical line and the line joining the center of the McGregor line and the sella turcica	[26]
Spino-cranial angle	The angle between the C7 slope and the straight line joining the middle of the C7 end plate and the middle of the sella turcica	[27]
C2-pelvic angle	The angle of a line from the C2 centroid to the femoral head (FH) and a line from the FH to the middle of the S1 endplate	[28]
Cervicothoracic pelvic angle	The angle of a line from the center of C2 to the FH and a line from the FH to center of T1	[28]
Craniocervical angle	The angle of the line from the center of C7 to the posterior corner of the hard palate and McGregor’s line	[28]
Occipitocervical inclination	The angle formed by the line connecting McGregor’s line and the posterior border of the C4 vertebral body	[29]
Clivo-axial angle	The angle subtended by lines drawn parallel to the dorsal surfaces of the clivus and dens	[30]
K-line tilt	The angle between the K-line and a line perpendicular to the horizon	[31]
C2 incidence angle	The angle between a line from the center of the FH through the midpoint of the sacral superior endplate and a line extended perpendicular to the C2 inferior endplate	[32]
C2 slope	The angle between the lower endplate of C2 and the horizontal plane	[33]

**Table 4:** Anterior fusion and posterior decompression for CSM/OPLL and cervical spine alignment.

1st Author	Year	Number of Patients (CSM/OPLL)	Definition of Cervical Deformity	Surgical Techniques (vs. Other Surgical Approach)	Risk Factors of Postoperative Malalignment	Prediction Factors of Postoperative Malalignment before Surgery	Risk Factors of Poor HRQOL Outcome
Zhou X [117]	2017	CSM: 67	-	ADF and LAMP (vs. LAMP only)	No difference	No difference	No difference
Yeh KT [118]	2015	DCM: 109	--	ADF and LAMP	-	-	-
Sun K [119]	2020	OPLL: 71	-	ADF (vs. laminectomy)	Laminectomy	-	Laminectomy
Li YC [120]	2022	OPLL: 55	-	ADF and laminectomy (vs. P-F)	-	-	-

CSM, cervical spondylotic myelopathy; OPLL, ossification of the posterior longitudinal ligament; HRQOL, health-related quality of life; ADF, anterior decompression with fusion; LAMP, laminoplasty; DCM, degenerative cervical myelopathy; P-F, posterior decompression with fixation.

## Data Availability

Not applicable.
